# Identification of hub genes associated with diabetic cardiomyopathy using integrated bioinformatics analysis

**DOI:** 10.1038/s41598-024-65773-z

**Published:** 2024-07-03

**Authors:** Hailong Cui, Die Hu, Jing Xu, Shuiying Zhao, Yi Song, Guijun Qin, Yanling Liu

**Affiliations:** 1 Department of Endocrinology and Metabolism, The First Affiliated Hospital of Zhengzhou University, Zhengzhou, 450052 China; 2 Academy of Medical Sciences of Zhengzhou University, Zhengzhou, 450052 China; 3 The Key Laboratory of Metabolism and Molecular Medicine of the Ministry of Education, Department of Endocrinology and Metabolism, Zhongshan Hospital, Fudan University, Shanghai, China

**Keywords:** Computational biology and bioinformatics, Cardiology, Endocrinology, Pathogenesis

## Abstract

Diabetic cardiomyopathy (DCM) is a common cardiovascular complication of diabetes, which may threaten the quality of life and shorten life expectancy in the diabetic population. However, the molecular mechanisms underlying the diabetes cardiomyopathy are not fully elucidated. We analyzed two datasets from Gene Expression Omnibus (GEO). Differentially expressed and weighted gene correlation network analysis (WGCNA) was used to screen key genes and molecules. Gene Ontology (GO), Kyoto Encyclopedia of Genes and Genomes (KEGG) enrichment analysis, and protein–protein interaction (PPI) network analysis were constructed to identify hub genes. The diagnostic value of the hub gene was evaluated using the receiver operating characteristic (ROC). Quantitative real-time PCR (RT-qPCR) was used to validate the hub genes. A total of 13 differentially co-expressed modules were selected by WGCNA and differential expression analysis. KEGG and GO analysis showed these DEGs were mainly enriched in lipid metabolism and myocardial hypertrophy pathway, cytomembrane, and mitochondrion. As a result, six genes were identified as hub genes. Finally, five genes (Pdk4, Lipe, Serpine1, Igf1r, and Bcl2l1) were found significantly changed in both the validation dataset and experimental mice with DCM. In conclusion, the present study identified five genes that may help provide novel targets for diagnosing and treating DCM.

## Introduction

Diabetic cardiomyopathy (DCM) is a prevalent cardiovascular complication of diabetes, characterized by hypertrophy, diastolic dysfunction, and intracellular lipid accumulation^1^. This condition significantly contributes to the high incidence and mortality of heart failure among individuals with diabetes.

There are several potential mechanisms through which diabetes causes cardiac dysfunction, including mitochondrial dysfunction, endoplasmic reticulum stress, oxidative stress, inflammation, myocardial fibrosis, and apoptosis^2–5^. However, the molecular mechanisms underlying the diabetes cardiomyopathy are not fully understood.

In recent years, bioinformatics technology has rapidly developed and become increasingly popular for exploring the molecular mechanisms of various diseases and investigating valuable biomarkers for diagnosis and treatment^6^. Bioinformatical studies have been proven effective in many metabolic diseases^7–10^, including diabetes and its complications. Therefore, integrated bioinformatics analysis may assist in probing the biomarkers and potential mechanisms of diabetes cardiomyopathy.

Traditional anti-heart failure or hypoglycemic therapy cannot effectively reverse or delay the progress of DCM, thus it is of great importance to search molecular biomarkers for the diagnosis and treatment of DCM. However, traditional methods of bioinformatics analysis sometimes overlook the interaction between genes and the relationship between genes and clinical features, leading to poor specificity of the final results. Weighted gene correlation network analysis (WGCNA) is a method that analyzes gene expression patterns of multiple samples. It clusters genes into modules with similar expression patterns and analyzes the relationship between modules and specific features, such as clinical characteristics of patients^11^. WGCNA provides a systematic insight into the associated networks and has become a reliable and effective tool for exploring hidden connections among numerous genes.

In this study, we downloaded gene expression matrices from the Gene Expression Omnibus (GEO) and examined the relationship between the expression of hub genes and diabetic cardiomyopathy using WGCNA, which analyzed the correlations among genes and the relationships between modules^12^. Additionally, Gene Ontology (GO) and Kyoto Encyclopedia of Genes and Genomes (KEGG) pathway enrichment analyses were performed to investigate the pathophysiologic feature of hub genes. Furthermore, a protein–protein interaction (PPI) network using Cytoscape was established for selected hub genes related to DCM. To validate the results obtained from the analysis, the accuracy and reliability of our analysis were further verified by establishing a mouse model with diabetic cardiomyopathy and detecting hub genes (Pdk4, Lipe, Igf1r, Serpine1, and Bcl2l1) through RT-qPCR to validate the results obtained from the analysis. The receptor-operated channels (ROC) analyses were conducted to confirm the sensitivity and specificity of these hub genes in DCM. In conclusion, our findings provide novel and reliable biomarkers for screening, diagnosis, and prognosis, as well as potential therapeutic targets for DCM. 

## Materials and methods

### Data collection and processing

**Figure 1:**
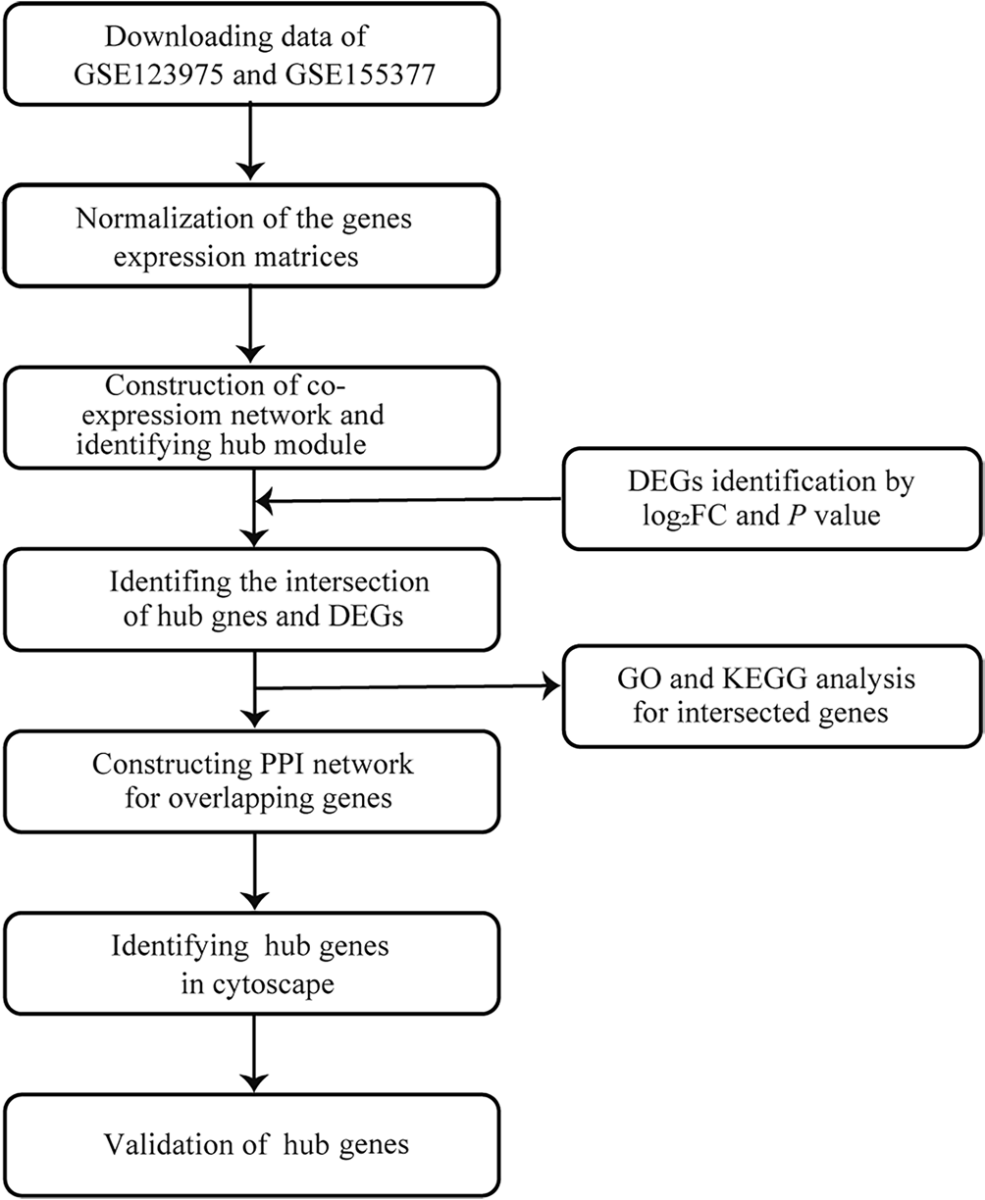
Flow diagram of the preparation, processing, analysis, and validation dates.

Data preparation, processing, analysis, and validation in the present study are shown in the workflow chart (Fig. 1).

Datasets GSE123975 and GSE155377 were downloaded from the GEO database (https://www.ncbi.nlm.nih.gov/geo/) for WGCNA and DEG analysis. The microarray data of GSE123975 was based on GPL10787 Platforms (Agilent-028005 SurePrint G3 Mouse GE 8x60K Microarray). GSE155377 was studied using the GPL23038 platform (Affymetrix Clariom S Assay, Mouse). The experimental protocol and details of the datasets are shown in Table 1.

**Table 1:** Characteristics of the included GEO datasets.

GEO_accession	Title	Protocol	Species	Platform	Type	Date
GSE123975	Enhanced Myocardial Glucose Delivery Accelerates the Development of Mitochondrial Dysfunction in the Diabetic Heart	Mice were made diabetic by using the low-dose streptozotocin (STZ) protocol as outlined by the Animal Models of Diabetic Complications Consortium. After 4–6 h of fasting, mice were injected with a stock solution of STZ (7.5 mg/mL; Axxora LLC, San Diego, CA) at 55 mg/kg of body weight or an equivalent volume of sodium citrate buffer (0.1 M, pH 4.5) for 5 consecutive days	Mouse	GPL10787	Array	2020.10.05
GSE155377	Angiotensin IV mitigates diabetic cardiomyopathy via downregulating FoxO1-mediated autophagy	Diabetes was induced in mice via intraperitoneal injection of streptozotocin for 5 days consecutively. Two weeks after STZ injection, mice with fasting blood glucose (FBG) ≥ 16.7 mM were chosen for subsequent experiments. In the NC group, mice were intraperitoneally injected with a vehicle daily for 5 days	Mouse	GPL23038	Array	2020.09.02

The R package inSilicoMerging was applied to dataset combination and normalization^13^. After merging the two GEO datasets, the batch effects were adjusted by empirical Bayesian methods^14^.

### Construction of the weighted co-expression network

The “WGCNA” package in R was used to construct co-expression network^15^. The top 2000 genes with maximum mean absolute deviation were selected into WGCNA to construct co-expression modules. At first, Bicor’s correlation matrices were calculated for all pair-wise genes. Then, a weighted adjacency matrix was constructed using a power function. As a soft-thresholding parameter, β could emphasize strong correlations between genes and penalize weak correlations. we first calculated the soft-thresholding power and adjacency. Next, the adjacency was transformed into a topological overlap matrix (TOM), which could measure the network connectivity of genes for further network generation. After that, we classify genes with similar expression profiles into colorful modules using a hierarchical clustering dendrogram of the TOM-based dissimilarity matrix (1-TOM)^16^. In addition, we correlated these module features with clinical features to investigate the co-expression network’s functional modules. As a result, the modules closely related to diabetes were subsequently selected for the next analyses. Genes that could not be classified into any module were finally placed into the grey module.

### Identification of clinically significant modules

The co-expression module is a collection of genes with high topological overlap similarity. Genes in the same module often have a higher degree of co-expression. In this study, we used two methods to identify the important modules relevant to clinical traits. Here, gene significance (GS) is defined as the absolute value of the correlation between the gene and the trait, and module membership (MM) represents the correlation of the genes with each module eigengene and clinical feature. Furthermore, module importance (MS) is defined as the correlation between the module eigengene and gene expression profile. Finally, we calculated the correlation between the modules and the clinical data to identify significant clinical modules.

### Identification of differentially expressed genes

To identify differentially expressed genes (DEGs) between diabetic and non-diabetic hearts, we performed the differential analysis of gene expression matrices from the merged matrices using the R package Limma (version 3.40.6)^17^. The cut-off value was set to |log_2_FC| ≥ 0.58, P-value < 0.05. The ggplot2 [3.3.6] R package was used to draw the volcano plot and heatmap of the DEGs. Then, the overlapping genes between DEGs and module genes were visualized by the R package VennDiagram^18^.

### Functional enrichment analysis

Overlapping genes were identified by taking the intersection of DEGs and genes in the key module. GO was used to identify characteristic biological attributes, including biological process (BP), cellular component (CC), and molecular function (MF). KEGG pathway enrichment analysis was performed to identify functional attributes^19^. The Database for Annotation, Visualization, and Integrated Discovery (DAVID) online database (http://david-d.ncifcrf.gov/) was used to perform GO and KEGG pathway analyses^20^. P < 0.05 was considered as statistically significant.

### Construction of protein–protein interaction networks and screening of hub genes

We used the Search Tool for the Retrieval of Interacting Genes (STRING) (https://string-db.org/) online database to construct protein–protein interaction (PPI) networks for overlapping genes. The PPI network was imported into Cytoscape software to visualize the PPI using the MMC, EPC, Degree, Betweenness, and DMNC algorithms in the CytoHuba plugin to filter the top 10 Hub genes. After taking the intersection of genes from different algorithms, the final results were visualized by ggplot2 [3.3.6] and a Venn diagram.

### ROC analysis

ROC curve was performed to assess the predictive accuracy of the prognostic signature by using pROC [1.18.0] and ggplot2 [3.3.6]. Other DCM models (GSE5606; GSE36875) were used for ROC analysis. The area under the curve (AUC) corresponding to these ROC curves was calculated to evaluate the performance of each model. An AUC > 0.7 indicated a good fitting effect.

### Animal experiments

Six-week-old male C57BL/6 mice were purchased from the Beijing Charles River Laboratory Animal Company (Beijing, China). All mice were housed at 21 ± 1 °C with 55 ± 10% humidity and a 12-h light/dark cycle. Mice were divided randomly into two groups, the DM group and the healthy control (HC) group. For the DM group, the C57BL/6J mouse model of type 1 diabetes was induced by intraperitoneal injection of streptozotocin (STZ), (50 mg/kg, Sigma-Aldrich) dissolved in 0.05 mM citrate buffer (pH 4.5) after 12 h of fasting for 5 consecutive days. On the third day after the last STZ injection, the blood glucose of the tail vein was measured and the random blood glucose ≥ 16.7 mmol/ L was considered to be successful. The HC group received an intraperitoneal injection with the same dose of citrate buffer. After 7 weeks of experiments, the mice were sacrificed at 12 weeks of age with an overdose of a mixture of xylazine and ketamine, and the heart tissues were immediately removed, frozen in liquid nitrogen, and stored at − 80 °C for further studies.

The laboratory animal production license number is SCXK (Yu) 2020-0008, the laboratory animal license number is SYXK (Yu) 2021-0009, and the experimental condition was a shielded environment. The study was approved by the institutional committee of the Animal Research Committee and Animal Ethics Committee of Henan Key Laboratory for Pharmacology of Liver Diseases (Approval number: 2019-44). The experimental protocols were performed following the guidelines of the Institutional Animal Care and Use Committee of Zhengzhou University. All methods were reported in accordance with the ARRIVE guidelines 2.0^21^.

### Quantitative RT-PCR analysis

Total RNA was extracted from heart tissues by RNAiso plus (Takara, Japan) and RNA was reversely transcribed to cDNA by Reverse transcription kit (Takara, Japan). Real-time PCR was performed using 10 μL of SYBR^®^ Green Realtime PCR Master Mix (beyotime, China) according to the manufacturer’s protocol on a Bio-Rad CFX 96 (Bio-Rad, USA) to detect the expression of Pdk4, Lipe, Igf1r, SERPINE, Bcl2l1, and β-actin as a normalizing control. The relative expression of these hub genes was performed using the 2^−ΔΔCT^ method. GraphPad Prism was used for statistical analysis, and t test was performed on the two groups of data that conformed to the normal distribution. P < 0.05 stands for the significance level. The primers sequence for Pdk4, Lipe, Igf1r, Serpine1, Bcl2l1and β-actin was listed in Table 2.

**Table 2:** Primers of genes determined by quantitative real-time polymerase chain reaction.

Gene symbol	Species	Forward primer	Reverse primer
Lipe	Mouse	CCCTACCTCAAGAACTGGGC	GCTCTCCAGTTGAACCAAGC
Igf1r	Mouse	CCGACGAGTGGAGAAATCTGT	GACTCGGAAGAGCAGCAAGT
Serpine1	Mouse	GCCACCGACTTCGGAGTAAA	TGAGCTGTGCCCTTCTCATT
Egf	Mouse	ATATTGACGAGTGCCAGCGG	GAGGGTGCGGTAGAGTCAGG
Bcl2l1	Mouse	GGCCTTTTTCTCCTTTGGCG	GATCCACAAAAGTGTCCCAGC
Pdk4	Mouse	CAGTGGACCCCGTTACCAAT	CACACTCAAAGGCATCTTGGAC

### Statistical analysis

Statistical software SPSS 23.0 was used to perform a *t*-test on the changes in qPCR results, and P < 0.05 was considered statistically significant.

## Results

### Dataset combination and normalization

**Figure 2:**
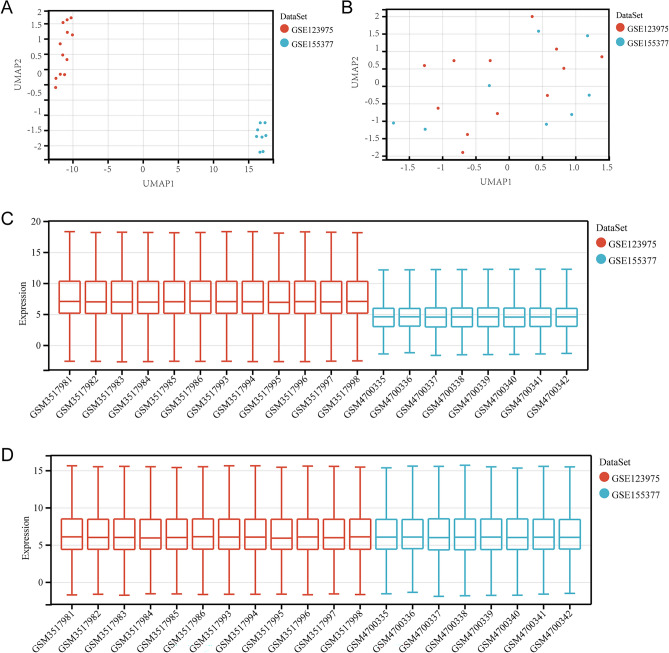
Datasets merging. (**A**,**B**) The UMAP and box plots before and after the removal of the inter-batch effect.

From the UMAP map, the samples of two datasets clustered separately before removing the batch effect (Fig. 2A). After adjusting the batch effect, samples were staggered (Fig. 2B). Boxplots also perform the same transformation from disarray to consistent, and the median was on a straight line (Fig. 2C,D) which shows the normalization of the dataset indicates a relatively high consistency.

### Weighted co-expression network construction and key modules identification

**Figure 3:**
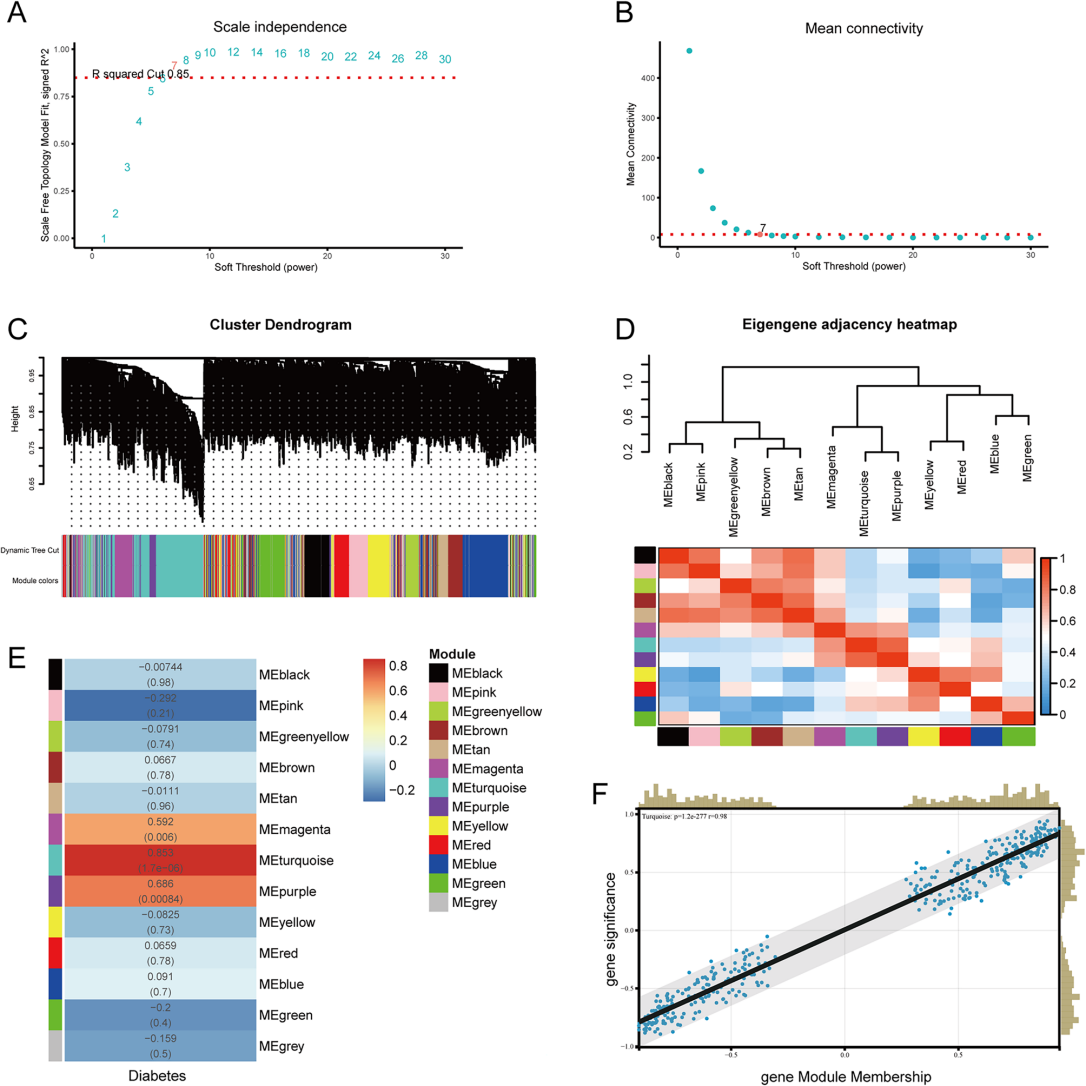
Identification of modules associated with diabetic cardiomyopathy using the weighted gene co-expression network analysis (WGCNA). (**A**) Analysis of the scale-free index for various soft-threshold powers (β). (**B**) Analysis of the mean connectivity for various soft-threshold powers. (**C**) Dendrogram of all differentially expressed genes clustered based on the measurement of dissimilarity (1-TOM). The color band shows the results obtained from the automatic single-block analysis. (**D**) Eigengene dendrogram and eigengene adjacency plot. (**E**) Heatmap of the correlation between the module eigengenes and clinical traits of diabetes. We selected the ME turquoise-grade block for subsequent analysis. *TOM* topological overlap matrix, *ME* module eigengene.

Using the “WGCNA” package in R, the DEGs with similar expression patterns were grouped into modules via the hierarchical cluster analysis. We set the soft threshold to 7 (R^2^ = 0.85) to construct a scale-free network (Fig. 3A,B), and a total of 13 modules were identified (Fig. 3C). Then, we clustered the eigengenes which can provide information about the relationship between pairwise the gene co-expression modules. The results showed that 12 modules (except for the grey module) could be clustered into 3 clusters. The heatmap of the co-expression modules shows a similar result (Fig. 3D). In addition, correlation plots between the module colors or genes and clinical traits were constructed (Fig. 3E). The module-trait heat map revealed the relationship between diabetes and genes within the modules. The turquoise module (374 genes) was significantly correlated with diabetic status (R = 0.853 and P = 1.7e−06). Also, the MM value was highly correlated in the turquoise module, suggesting that the genes in this module were probably related to the disease status (Fig. 3F). Above all, the turquoise module was highly related to pathological grades of diabetes, thus this module was selected as a clinically important module for further analysis.

### Identification of DEGs and overlapping genes

**Figure 4:**
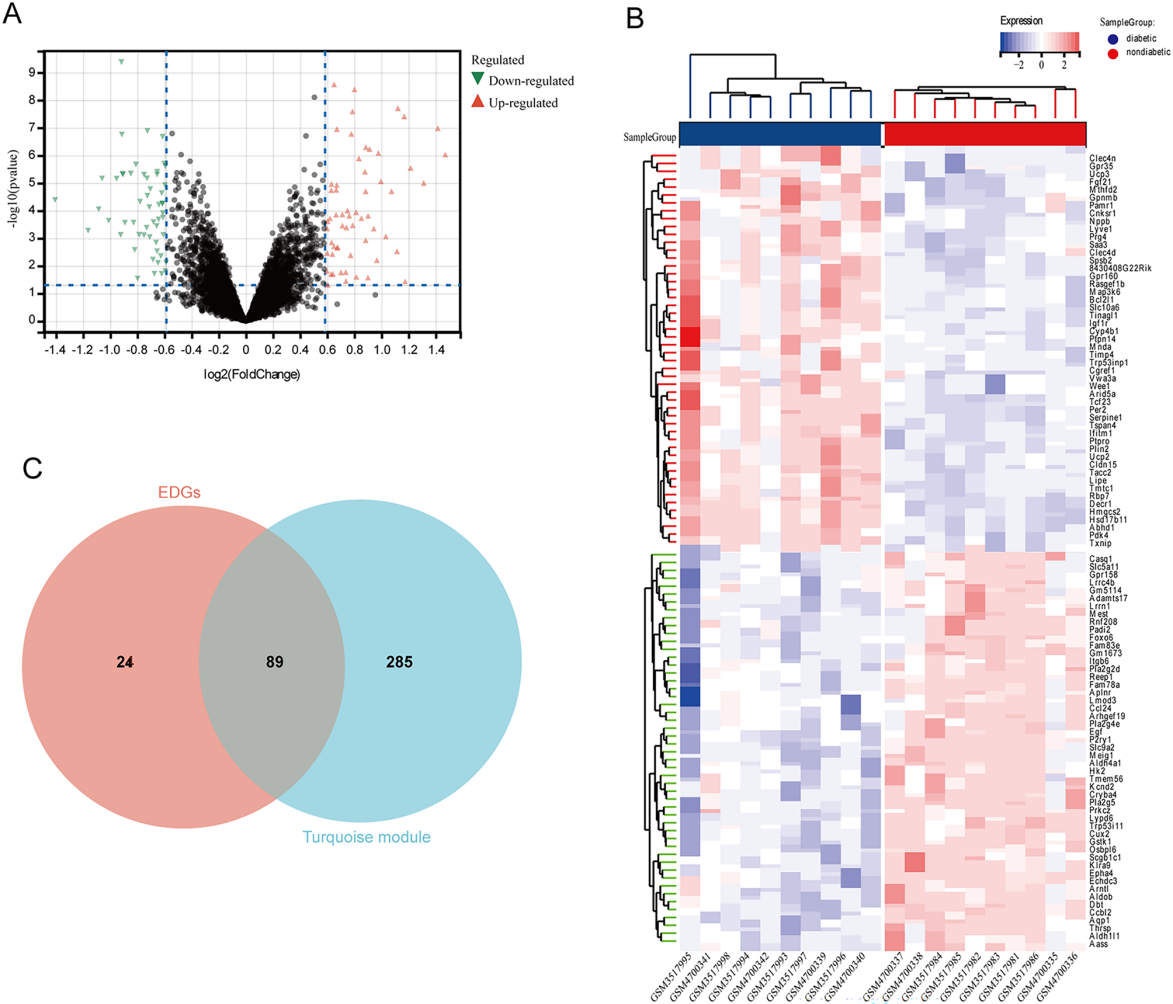
Differentially expressed genes (DEGs) in the merged datasets. (**A**) Volcano plot. (**B**) Heatmap plot of top 100 DEGs. (**C**) The Venn diagram of genes from the DEGs and turquoise module.

Through the Limma (R package) analysis tool, we finally identified a total of 113 DEGs including 60 upregulated genes and 53 downregulated genes. DEG selection criteria were set at P < 0.05 and |log_2_FC| ≥ 0.59. The visualization of these DEGs is shown by the volcano plot (Fig. 4A). The top 100 DEGs were visualized by a heatmap which suggests a great difference in transcriptional profile (Fig. 4B). 89 genes were selected from the intersection of turquoise module genes and DEGs by the method of Venn diagram online drawing tool (Fig. 4C).

### Functional enrichment analysis of DEGs

**Figure 5:**
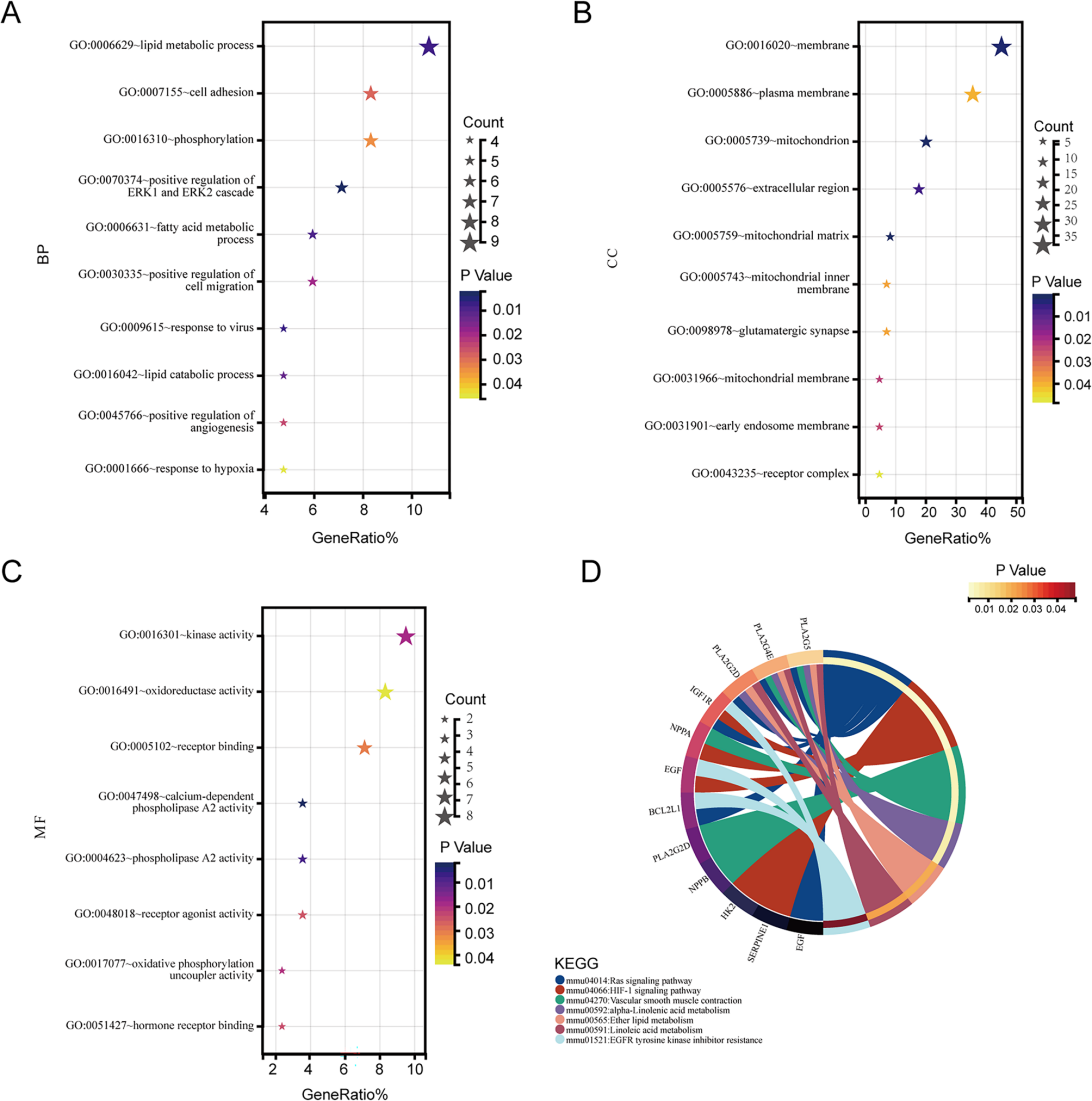
Functional annotation of the 89 overlapping genes. (**A**) Biological processes (BP). (**B**) Cell composition (CC). (**C**) Molecular function (MF). (**D**) KEGG analysis.

Using GO analysis and KEGG analysis in the DAVID online database, we performed an enrichment analysis of the biological significance of these 89 DEGs. BP analysis revealed that these 89 DEGs were significantly enriched in the lipid metabolic process, cell adhesion, phosphorylation, positive regulation of ERK1 and ERK2 cascade, fatty acid metabolic process, positive regulation of cell migration, response to virus and lipid catabolic process (Fig. 5A). Enriched CC terms were mainly involved in membrane and mitochondrion (Fig. 5B). MF showed that these genes were highly related to kinase activity, oxidoreductase activity, receptor binding, calcium-dependent phospholipase A2 activity, phospholipase A2 activity, and receptor agonist activity (Fig. 5C). KEGG analysis established that markedly enriched pathways for the hub genes included Ras signaling pathway, HIF1 signaling pathway, vascular smooth muscle contraction, alpha-linolenic acid metabolism, ether lipid metabolism, linoleic acid metabolism, EGFR tyrosine kinase inhibitor resistance (Fig. 5D).

### PPI network construction and hub genes identification

**Figure 6:**
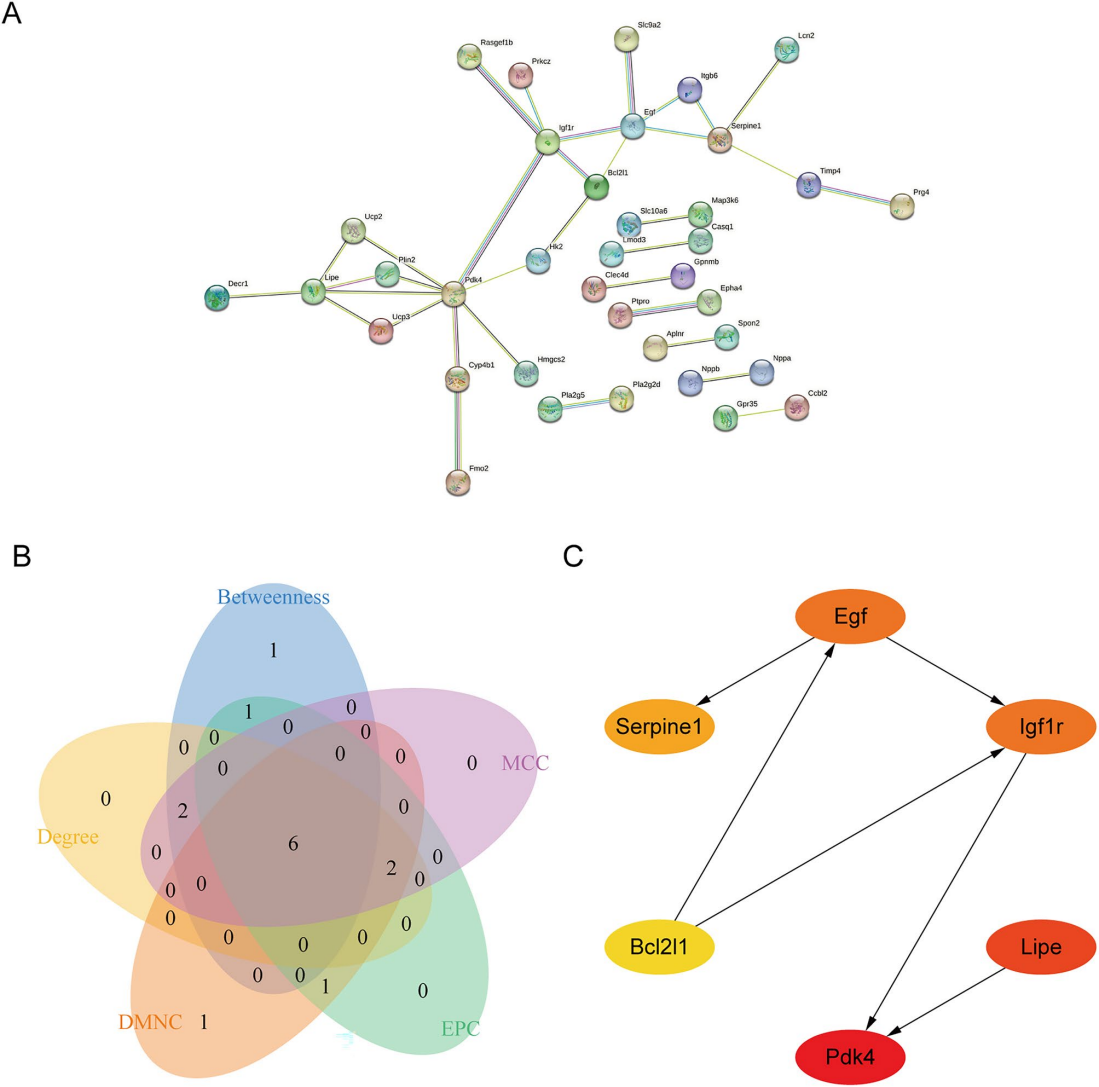
PPI network and identification of hub genes. (**A**) PPI network of the genes between DEG lists and turquoise module constructed by STRING. The nodes represent the genes. Edges indicate interaction associations between nodes. (**B**) Identification of 6 candidates for hub genes by four algorithms. (**C**) PPI network of the hub genes. PPI, protein–protein interaction; DEG, differentially expressed genes; STRING, Search Tool for the Retrieval of Interacting Genes.

To establish the interactions of these DEGs in DCM, we used the STRING (https://www.string-db.org/) online database to construct a PPI network. Results were visualized as the PPI network by the Cytoscape software (Fig. 6A). Venn diagram showed the intersecting genes among the six algorithms (Fig. 6B). The PPI network of the 6 overlapping genes including Pdk4, Lipe, Egf, Igf1r, Serpine1, and Bcl2l1 was shown in Fig. 6C.

### Validation of the expression levels of hub genes in vivo

**Figure 7:**
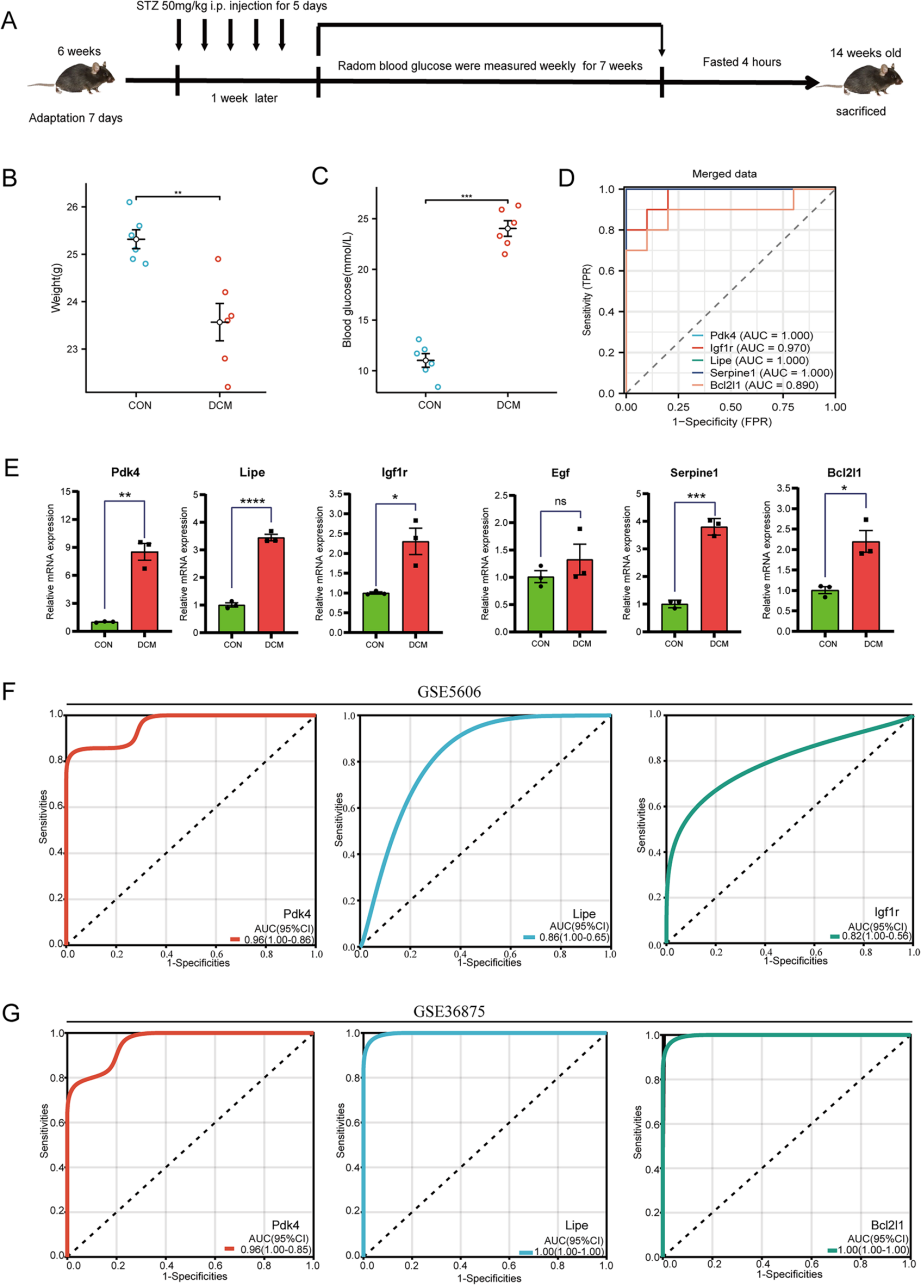
Establishment of diabetic cardiomyopathy mouse model and validation of hub genes and ROC analysis. Schematic illustration of diabetic cardiomyopathy mouse model. (**B**,**C**) Body weight, and random blood glucose, at 7 weeks after STZ injection (n = 6 each). (**D**) ROC curve analysis of Pdk4, Igf1r, Lipe, Serpine1 and Bcl2l1 in the merged dataset. (**E**) Gene expression in a mouse model of DCM and Con. ****P < 0.0001, ***P < 0.001, **P < 0.01, *P < 0.05. (**F**) ROC curve analysis of Pdk4, Igf1r, Lipe, Serpine1 and Bcl2l1 in the validating dataset GSE5606. (**G**) ROC curve analysis of Pdk4, Igf1r, Lipe, Serpine1 and Bcl2l1 in the validating dataset GSE36875.

As described in *Materials and Methods*, we used a mouse model of persistent hyperglycemia induced by STZ (Fig. 7A). After the STZ injection, random blood glucose and body weight were measured. The diabetic mice exhibited a significant reduction in body weight compared with the control group at 7 weeks after STZ injection (Fig. 7B). Random blood glucose and heart weight/body weight were significantly increased in the diabetic group compared with the control group (Fig. 7C).

RNA was extracted from heart tissues for quantitative real-time PCR analysis. An unpaired *t*-test was used for comparison between the two groups. The results suggested that 5 hub genes [Pdk4 (t = 8.312, P = 0.0011), Lipe (t = 17.61, P < 0.0001), Igf1r (t = 3.906, P = 0.0175), Serpine1 (t = 14.73, P = 0.0001), Bcl2l1 (t = 4.246, P = 0.0001), P = 0.0132)] were significantly upregulated in diabetic group compared with Control except for Egf (t = 1.04, P = 0.3571) (Fig. 7E). The expression levels of hub genes in vivo were consistent with the analysis above (Table 3).

**Table 3:** Expression pattern of potential hub genes in merged dataset.

Gene symbol	Full name	log_2_FC	*P* value	Regulation
Pdk4	Pyruvate Dehydrogenase Kinase 4	1.713454512	3.91E−09	Up
Lipe	Lipase E	0.912352814	0.000000592	Up
Igf1r	Insulin-Like Growth Factor 1 Receptor	0.620589596	0.000190791	Up
Egf	Epidermal Growth Factor	− 0.73106055	0.000413103	Down
Serpine1	Serpin Family E Member 1	1.415859492	0.000000104	Up
Bcl2l1	BCL2 Like 1	0.633201268	0.001409574	Up

### Diagnostic significance of DEGs

**Figure 8:**
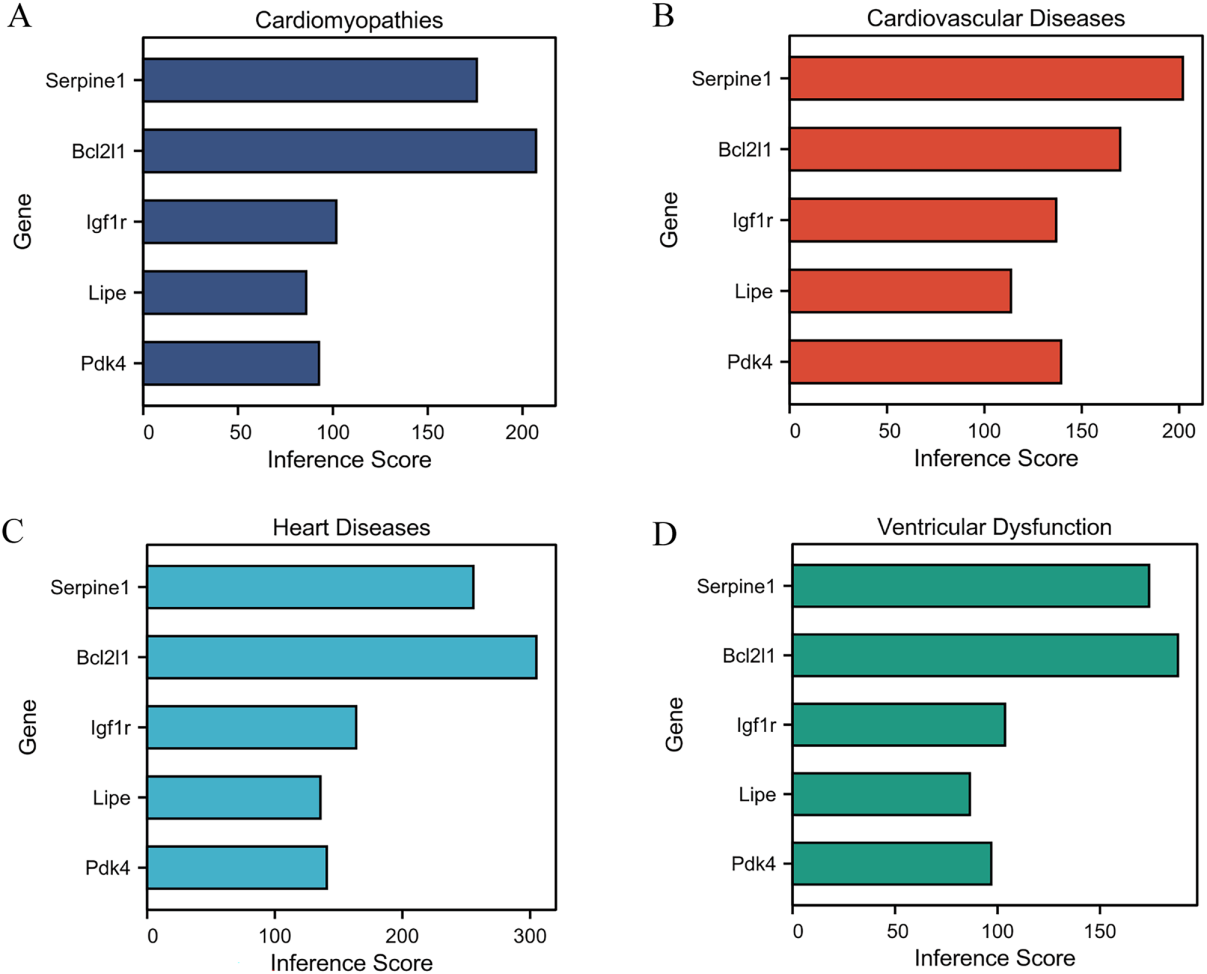
Relationship analysis of the five potential hub genes with cardiovascular and diseases in CTD. Five potential hub genes (Pdk4, Igf1r, Lipe, Serpine1 and Bcl2l1.) targeted multiple cardiovascular diseases, including (**A**) cardiomyopathies, (**B**) cardiovascular disease, (**C**) heart disease, (**D**) ventricular dysfunction.

To determine which DEGs have diagnostic significance for DCM patients, the ROC analyses were conducted to explore the sensitivity and specificity of DEGs for DCM diagnosis. All the five genes (Pdk4, Lipe, Igf1r, Serpine1, and Bcl2l1) exhibited prospective diagnostic values and ROC curves of these hub genes have been depicted in Fig. 7D,F,G. The results showed that Pdk4 (AUC = 1.000 in merged data; 0.959 in GSE5606; 0.960 in GSE36875) and Lipe (AUC = 1.000 in merged data; 0.857 in GSE5606; 1.000 in GSE36875) have best diagnostic values for differentiating DCM from healthy control. The hub genes in this study may act as biomarkers to estimate the development of DCM and verify the effectiveness of the treatment of DCM. Moreover, the relationship analysis of the five potential genes associated with DCM was performed using the online CTD database. The results suggested genes (Pdk4, Igf1r, Lipe, Serpine1, and Bcl2l1) targeted cardiovascular diseases including cardiomyopathies, ventricular dysfunction, etc. (Fig. 8).

## Discussion

DCM is a cardiac dysfunction that affects approximately 12% of diabetic patients, leading to heart failure and death^22^. However, the lack of an efficient and specific diagnostic method for DCM may be attributed to the unclear molecular mechanisms involved in its pathophysiology and its asymptomatic for many years. Therefore, it is crucial to explore susceptibility genes and identify molecular biomarkers for early diagnosis, prevention, and personalized therapy in DCM.

With advancements in DNA sequencing and chip technology, bioinformatics methods have been widely employed to study various metabolic diseases. In this study, we utilized multiple bioinformatics methods to investigate the potential biological mechanisms of DCM.

We applied WGCNA to analyze the datasets GSE123975 and GSE155377, and compared the DEGs profiles of DCM obtained from the GEO database. We identified 60 upregulated DEGs and 53 downregulated DEGs. Finally, we selected 89 DEGs between the diabetic and nondiabetic groups. 

Furthermore, we performed GO term enrichment and signaling pathway enrichment analyses to further explore these DEGs. The BP annotation revealed enrichment in lipid metabolism, cell adhesion/migration, positive regulation of ERK1 and ERK2 cascade, fatty acid metabolic process, etc. Previous studies have reported that diabetic dyslipidemia and intramyocardial lipid accumulation are the key pathological features of DCM, triggering cellular signaling and modifications of proteins and lipids through toxic metabolic intermediates^23,24^. Extracellular signal-regulated kinase 1/2 (ERK1/2) has been implicated in myocardial dysfunction, particularly in DCM^25^. Cell adhesion and migration are involved in the process of myocardial fibrosis, contributing to severe cardiac dysfunction associated with DCM. Maintaining the bioenergy function of mitochondria is crucial for cardiac function in diabetic patient^26^. MF analysis revealed the activation of kinases, oxidoreductase, phospholipase A2, etc., during the development of cardiomyopathy in diabetic patients. KEGG pathway analyses identified the Ras signaling pathway, HIF1 signaling pathway, vascular smooth muscle contraction, alpha-linolenic acid metabolism, ether lipid metabolism, linoleic acid metabolism, and EGFR tyrosine kinase inhibitor resistance as important pathways in DCM. The Ras and HIF1 signaling pathway closely associated with oxidative stress and cell apoptosis, have been found to participate in the pathogenesis of DCM^27–30^. Dysregulation of vascular smooth muscle contraction leads to cardiac dysfunction in diabetes^31^. Additionally, the adverse cardiac effects of lipotoxicity have been mentioned earlier.

We further investigated hub genes by constructing a PPI network to explore the crucial genes involved in DCM development. We find that Pdk4, Lipe, Igf1r, Serpine1, and BCl2l1 may play key roles in the incidence and prognosis of DCM.

Pdk4 (pyruvate dehydrogenase kinase 4) belongs to the pyruvate dehydrogenase kinases (PDKs) family, which are mitochondrial enzymes that suppress the conversion of pyruvate to acetyl CoA via inhibitory phosphorylation of the pyruvate dehydrogenase complex (PDC)^32^. PDK4 is critically involved in the regulation of the cellular energetic metabolism in the heart and skeletal muscle. Previous studies have shown a close association between Pdk4 and diabetes and heart disease^33^. In diabetes, Pdk4 activity is markedly upregulated^34,35^. Zhao et al.^36^ demonstrated that cardiac-specific overexpression of Pdk4 leads to a loss of metabolic flexibility that exacerbates cardiomyopathy. Myocardial metabolic stress is an important factor underlying diabetic cardiac dysfunction^37^. A recent study by Reichelt et al.^38^. also predicted that high fructose-induced myocardial TG and glycogen accumulation are attributable to increased Pdk4 activity. Furthermore, Pdk4 was identified as a susceptibility gene for diabetic cardiomyopathy in previous research^39^.

Lipe (lipase E, hormone-sensitive type) encodes the hormone-sensitive lipase (HSL), a key enzyme that is involved in the hydrolysis of intracellular triglycerides and lipolysis, releasing fatty acids as an energy substrate in adipose tissues^40,41^; Dysregulation of HSL expression and activity has been observed in disorders, such as obesity and T2DM, indicating its significance in adipocyte function and regulation of systemic lipid and glucose homeostais^42^. Defective insulin secretion in response to glucose has been reported in β-cells from Lipe null mice^43^. Another study found that cardiac HSL plays a role in controlling the accumulation of triglyceride droplets and may affect the expression of cardiac genes related to cardiac dysfunction^44^. Moreover, Lipe has been analyzed as a candidate gene for the development of type 2 diabetes in a community-based sample of American Indians^45^.

Insulin-like growth factor 1 receptor (Igf1r) belongs to the family of tyrosine kinase receptors^46^. Ligand binding (generally with IGF1) induces phosphorylation of specific tyrosine residues of Igf1r^47^. Igf1r is expressed in many cells throughout the body, including the brain and heart. It has been established that Igf1r signaling plays a crucial role in regulating energy metabolism and glucose homeostasis, making it a master regulator of pathophysiological processes related to T2D^48,49^. In the development of T2D, Igf1r is found overexpressed or activated in response to hyperglycemia and hyperinsulinemia, leading to the deterioration of the disease^50^. Studies have shown that Igf1r-deficient brown adipocytes exhibit increased insulin sensitivity^51^. Furthermore, Abdellatif et al. reported that failing hearts displayed exaggerated Igf1r expression and signaling activity, which is recognized to be critical for cardiac homeostasis^52^. A vascular injury that is induced by hypercholesterolemia also increases Igf1r signaling, stimulating smooth muscle cell migration and division and contributing to accelerated atherosclerosis^53^. The evolutionarily related insulin and insulin-like growth factor-1 (IGF-1) axes are implicated in the development of cardiovascular events^54^. Moreover, the overexpression of Igf1r in the myocardium of diabetic rats has been reported, suggesting the potential of Igf1r as a diagnostic marker for DCM.

Serine protease inhibitor clade E member 1 (Serpine1) belongs to the Serine protease inhibitor family^55^. It plays a key role in modulating the plasminogen/plasminase system and has been associated with various conditions, including cardiovascular diseases, inflammation, cancer, metabolic disorders, aging, tissue fibrosis, etc. Plasminogen activator inhibitor (PAI)-1, a member of the Serpine1 superfamily, regulates the fibrinolytic process, and its levels are elevated in diabetes and insulin-resistant states^56^. Hyperinsulinemia and hyperglycemia in T2D patients have been shown to increase PAI-1 levels, resulting in hypofibrinolysis, pathological fibrin deposition, and heart damage^57^. Considering that fibrosis is a predominant feature of ventricular remodeling and DCM, Serpine1 (PAI-1) may serve as a promising biomarker for predicting the risk of cardiac complications in diabetic patients.

Bcl2l1, also known as B cell lymphoma 2 like 1, is a member of the family of Bcl-2 family. This family of proteins plays a role in regulating apoptosis by controlling the release of pro-apoptotic factors from the mitochondrion^58^. One special protein encoded by Bcl2l1, called Bcl-xL, is well known for its anti-apoptosis abilities. It has been demonstrated by Sakuma et al. that there is an increase in the expression of Bcl-xL induced by high glucose levels, which may be important in the development of atherosclerosis in diabetic patients^59^. Additionally, Bcl-xL has been shown to inhibit apoptosis and protect the heart against ischemia/reperfusion injury.

However, the special relationship between Bcl2l1 and DCM remains poorly understood. Based on previous studies, we can hypothesize that Bcl2l1 may be upregulated in response to the adverse effects of a high glucose environment on the myocardium in patients with diabetes, as a reactive mechanism to resist these effects.

Overall, this study has made significant contributions to our understanding of the biological processes involved in DCM through the use of comprehensive bioinformatics analysis and the identification of key genes. The utilization of the WGCNA method has provided a reliable and effective approach for diagnosing and treating DCM.

It is important to acknowledge the limitations of this study. Firstly, although the hub genes were generated from two different datasets, further validation in a larger patient cohort is necessary to strengthen the reliability of these findings. Additionally, the relatively small sample size in this study may have weakened the conclusiveness of the results. To address this limitation, further studies should aim to conduct clinical experiments that demonstrate the mechanistic connections between these identified genes and DCM. This would help provide more robust evidence for their role in the pathological process of DCM.

In conclusion, this study has successfully identified five hub genes (Pdk4, Lipe, Igf1r, Serpine1l, and Bcl2l1) that are potentially involved in the occurrence and development of DCM. The RT-qPCR verification in mice with diabetic cardiomyopathy confirmed the statistically increased mRNA expression of these genes. These findings suggest that these hub genes may serve as novel and reliable biomarkers for screening, diagnosis, and prognosis of DCM, as well as potential therapeutic targets for DCM.

## Data Availability

The datasets generated and analyzed in our study are available in the public Gene Expression Omnibus database (https://www.ncbi.nlm.nih.gov/geo/query/acc.cgi?acc=GSE123975; https://www.ncbi.nlm.nih.gov/geo/query/acc.cgi?acc=GSE155377; https://www.ncbi.nlm.nih.gov/geo/query/acc.cgi?acc=GSE5606; https://www.ncbi.nlm.nih.gov/geo/query/acc.cgi?acc=GSE36875). Further inquiries can be directed to the corresponding author.

## Abbreviations

BP: Biological process
CC: Cellular component
CCl4: Carbon tetrachloride
DAVID: Database for Annotation, Visualization and Integrated Discovery
DEG: Differentially expressed genes
FC: Fold change
FDR: False discovery rate
GEO: Gene Expression Omnibus
GO: Gene Ontology
HC: Healthy controls
HCC: Hepatocellular carcinoma
HFD: High-fat diet
IGF1: Insulin-like growth factor 1
IGF2: Insulin-like growth factor 2
IGFBP2: Insulin-like growth factor binding protein 2
JNK: C-Jun N-terminal kinase
JUN: Jun proto-oncogene
KEGG: Kyoto Encyclopedia of Genes and Genomes
MCC: Maximal Clique Centrality
MF: Molecular function
NAFLD: Non-alcoholic fatty liver disease
NASH: Non-alcoholic steatohepatitis
NAFL: Non-alcoholic fatty liver
PPI: Protein-protein interaction
Serpine1: Serpin family E member 1
STRING: Search Tool for the Retrieval of Interacting Genes
TOM: Topological overlap matrix
WGCNA: Weighted gene co-expression network analysis
